# Characterization and susceptibility of streptococci and enterococci isolated from Nile tilapia (*Oreochromis niloticus*) showing septicaemia in aquaculture and wild sites in Egypt

**DOI:** 10.1186/s12917-017-1289-8

**Published:** 2017-11-25

**Authors:** Kamelia M. Osman, Khalid S. Al-Maary, Aymen S. Mubarak, Turki M. Dawoud, Ihab M. I. Moussa, Mai D. S. Ibrahim, Ashgan M. Hessain, Ahmed Orabi, Nehal M. Fawzy

**Affiliations:** 1 Department of Microbiology, Faculty of Veterinary Medicine, Cairo University, Giza, Egypt; 2 Department of Botany and Microbiology, College of Science, King Saud University, Riyadh, Kingdom of Saudi Arabia; 3 Department of Public Health, College of Applied Medical Science, King Khalid University, Abha, Kingdom of Saudi Arabia; 4 Department of Health Science, College of Applied Studies and Community Service, King Saud University, Riyadh, Kingdom of Saudi Arabia; 5 Department of Fish Diseases and Management, Animal Health Research Institute, Dokki, Giza, Egypt

**Keywords:** *Streptococcus* species, Tilapia (*Oreochromis* spp.), Lancefield C, *L. Garvieae*, *S. Dysgalactiae*, *L. Mesenteroides cremoris*, Antibiotic resistance

## Abstract

**Background:**

The present investigation was an endeavor into the elucidation of the disease-causing pathogen of streptococcosis in Nile tilapia (*Oreochromis niloticus*) in Egypt affecting adult fish cultured and wild fish in the Nile river. Fish were obtained from commercial fishermen, collected as part of their routine fishing activities. The researchers observed the routine fishing process and selected fish for use in the study, at the point of purchase from the fisherman.

**Results:**

Diseased fish showed exophthalmia with accumulation of purulent and haemorrhagic fluid around eyes, and ventral petechial haemorrhages. The Post mortem examination revealed, abdominal fat haemorrhage, pericarditis and enlargement of the liver, spleen and kidney. Gram-stained smears revealed the presence of Gram-positive cocci, β-hemolytic, oxidase and catalase negative. Analysis of the 16S rRNA gene confirmed that the 17 tilapia isolates studied were 6/17 *Enterococcus faecalis,* 2/17 *Enterococcus gallinarum,* 3/17 *Streptococcus pluranimalium,* 2/17 *Aerococcus viridans,* 1/17 isolate of each *Streptococcus dysgalactiae, Streptococcus anginosus, Lactococcus garvieae* and *Granulicetella elegans*/*Leuconostoc mesenteroides cremoris*. It should be noted that there was no mixed infection. Multiple resistance was observed and the most frequent antibiotic combination was penicillin, ampicillin, vancomycin, chloramphenicol, rifampicin, ofloxacin, clindamycin, erythromycin and tetracycline representing eight classes.

**Conclusions:**

Consequently, we concluded that *Streptococcus* species are an emerging pathogen for Nile tilapia aquaculture in Egypt and to be considered as a new candidate in the warm water fish diseases in Egypt with special reference to *L. garvieae, S. dysgalactiae* in addition to *L. mesenteroides cremoris* which was not reported before from tilapia and taking into consideration their zoonotic implications for public health.

## Background

The common name Nile tilapia (*Oreochromis niloticus*) is a species of Tilapia (*Oreochromis* sp.) which in turn is the regular name for about a hundred species of cichlid fish from the tilapiine cichlid lineage [1]. They are freshwater fish species, local to Africa and Middle East areas [2], and the third biggest group of bony fish. Delineations from Egyptian tombs underscored that Nile tilapia have been developed for more than 4000 years [3]. By the second half from the twentieth century, tilapia were brought into a myriad of tropical, sub-tropical and mild locales of the globe [4] becoming a standout asset amongst the most refined fish species adding to the global nourishment in the fish industry [5] where the sustenance of more than half a billion people in the Third World are supported by fisheries and aquaculture [6]. The market price of farmed tilapia boosted up from 154 million USD in 1984 to 4000 million USD in 2010 [7]. China, with a production capacity of 806,000 MT represents almost 50% of the total global production positioned itself as the largest Nile tilapia producer, with Egypt to follow with 200,000 MT, the Philippines with 111,000 MT, Thailand with 97,000 MT and Indonesia with 72,000 MT. Five other countries come next in their capacity for Nile tilapia production, The Lao People’s Democratic Republic, Costa Rica, Ecuador, Colombia and Honduras [8]. Also, although significant quantities of tilapia are also produced annually by Cuba, Israel, Malaysia, the USA, Viet Nam and Zimbabwe, yet it should be noted that, their production is reported to FAO as ‘tilapias nei’ (which may include other tilapia species) and ‘freshwater fishes nei’ [8].

Streptococcosis is a worldwide threat and epidemiological studies revealed 500 streptococcal isolates in more than 50 sites in 13 countries (http://www.thefishsite.com/articles/190/streptococcus-in-tilapia/#sthash.G4T0XzsI.dpuf). Streptococcosis, also known as ‘pop-eye’, is contagious with high mortality and has assumed its importance due to being the most crushing threat as it can bring about huge number of deaths of large size fish causing heavy commercial losses in Australia, Israel, Italy, Japan, Korea, South Africa, Colombia, Indonesia and USA [9, 10]. The global commercial losses estimated to reach 250 million USD in 2008 [11]. Within 3 to 7 days, acute *Streptococcus* infections in fish induce >50% mortality rates [12] while chronic infections the mortalities could extend to several weeks, with a daily death of one or two of the fish. In most cases, the clinical symptoms of *Streptococcus* infection, with no species differences (http://www.thefishsite.com/articles/190/streptococcus-in-tilapia/#sthash.G4T0XzsI.dpuf), is usually in the form of lethargic, erratic swimming (spiraling or spinning swimming), dark skin pigmentation, exophthalmia with opacity and haemorrhage in the eye, abdominal distension, diffused haemorrhage in the operculum, around the mouth, anus and base of the fins and enlarged blackened spleen [8, 10, 13–15].

Streptococcosis is not limited to geographic boundaries or/and host range causing a global flare-up in aquaculture farms [16]. A diversity of fish species are susceptible to infection, including sturgeon, various ornamental fish, including rainbow sharks, red-tailed black sharks, rosey barbs, danios, some cichlids including Venustus (*Nimbochromis* (“Haplochromis”) *venustus*) and *Pelvicachromis* sp. and several species of tetras, coho salmon, yellowtail (*Seriola quinqueradiata*), Jacopever *(Sebastes schlegeli*), salmonids, Japanese eel (*Anguilla japonica*), ayu and tilapia (*Oreochromis* spp.), striped bass (*Morone saxatilis*), Atlantic croaker (*Micropogon undulatus),* blue fish (*Pomatomus saltatrix*), channel cat fish, golden shiner (*Notemigonous chrysoleuca*), hardhead (sea) cat fish (*Arius felis*), menhaden (*Brevoortia patronus*), pin fish (*Lagodon rhomboides*), sea trout (*Cynoscion regalis*), silver trout (*Cynoscion nothus*), spot (*Leiostomus xanthurus*), stingray (*Dasyatis* sp.), striped bass (*Morone saxatilis*), striped mullet (*Mugil cephalus*) and turbot (*Scophthalmus maximus*) [17, 18].

The most relevant *Streptococcus* species that cause disease in the tilapia farming globally are *S. iniae*, *S. agalactiae, S. dysgalactiae* and *Lactococcus garviae* [19, 20]. Since the first description of a streptococcal infection in rainbow trout (*Oncorhynchus mykiss*) [21], the diversity and the inherent characteristics of the bacterial species incriminated with streptococcosis was debatable [9, 17]. High and low temperatures affect the virulence agents at which streptococcosis is induced [22]. Warm water streptococcosis implicates species such as *L. garvieae* (synonym *E. seriolicida*), *S. iniae* (synonym *S. shiloi*), *S. agalactieae* (synonym *S. difficilis*) or *S. parauberis* inducing mortalities at temperatures above 15 °C. Differently, *Vagococcus salmoninarum* or *L. piscium* and *S. phocae* are incriminated in cold water streptococcosis at temperatures below 15 °C. The ability of *S. phocae* to cause cold water streptococcosis was evident when researchers were able to isolate it from marine mammals inhabiting cold waters, including Cape fur seal (*Arctocephalus pusillus pusillus*), ringed seal (*Phoca hispida*) and harbor porpoise (*Phocoena phocoena*) and gray seal (*Halichoerus grypus*), harbor seal (*Phoca vitulina*) and cetaceans [9, 10]. Pathogenic fish *Streptococcus* species have been associated with *S. agalactiae, S. difficilis, S. dysgalactiae, S. equi, S. equisimilis, S.* (*= E*.) *faecium, S. ictaluri, S. iniae, S. milleri, S. parauberis, S. phocae, S. pyogenes* and *S. zooepidemicus.* In addition, *E. faecalis* NCTC 775 T, *E. faecium* NCTC 7171 T, *L. lactis* NCFB 604, *S. mutans* NCFB 2062 provoke streptococcosis in Atlantic salmon and rainbow trout [9, 17].

Zoonotically, streptococcicosis is of great significance to delineate the infectious etiology of streptococcosis in Nile tilapia as a potential cause of disease in humans [16, 17] which caused a significant increase in the use of antibiotics in aquaculture. The present investigation was an endeavor into the elucidation of the disease-causing pathogen of streptococcosis in Nile tilapia (*O. niloticus*) in Egypt affecting adult fish cultured and wild fish in the Nile river by studying the phenotypic, antibiotic resistance and molecular characterization of the streptococcal strains isolated from Nile tilapia demonstrating septicemia.

## Methods

### Fish sampling

**Fig. 1:**
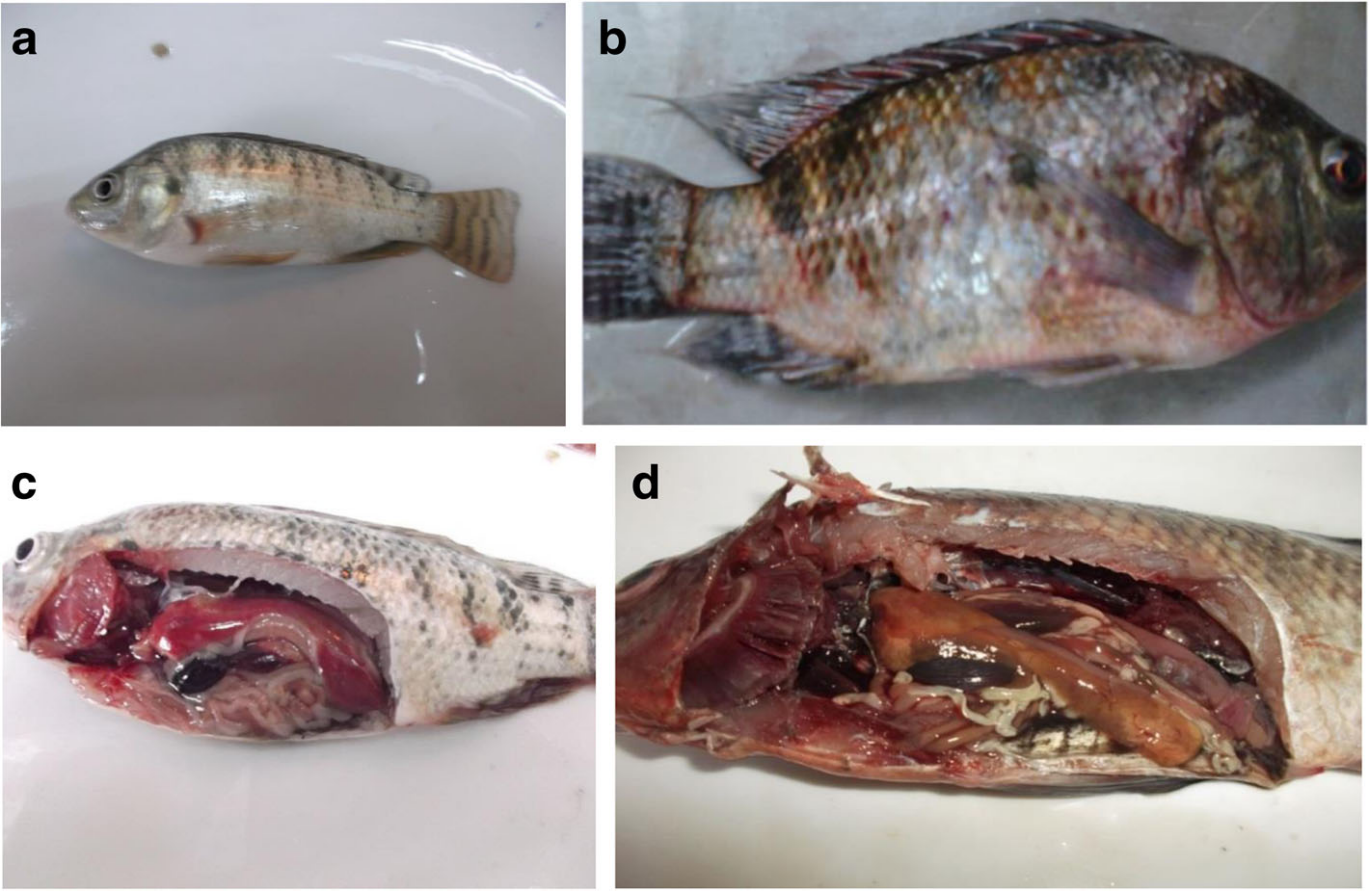
**a** Body shape of Tilapia fish laterally compressed to oval. Lateral line interrupted with cycloid scales. Caudal fin has distinct vertical stripes and strong body vertical bands. Healthy transparently clear and spherical eye lenses. Normal glistening skin with no pathological lesions observed. **b** Tilapia fish showing externally eye redness, hemorrhage and ulcers on different parts of the body, hemorrhagic dorsal and tail fins, extensively detached scales and distended abdomen with signs of bleeding beneath the skin appearing as small dots the size of a pinprick or as a patch. **c** Shows normal appearance of Tilapia fish with normal liver, kidney, gall bladder and intestines with bright red gills. **d** Tilapia fish postmortum internal findings revealed tipical signs of septicemia in which severely congested gill, kidney, spleen, intestine and heart, dark gall bladder, in addition to hemorrhagic ascites, friable pale liver, muscle redness, and parts of the intestine were devoid of food

Fish were obtained from commercial fishermen, collected as part of their routine fishing activities. The researchers observed the routine fishing process and selected fish for use in the study, at the point of purchase from the fisherman the fish were deceased and as such no ethical approval was required for this study. A total number of 80 Nile tilapia were collected as freshly dead or moribund fish (Fig. 1a-d) presenting at least one or more of the clinical signs of septicaemia (eye lesions: in the form of unilateral or bilateral eye redness/opacity, skin lesions: detached scales, extensive skin congestion, ulcers, hemorrhage, or dark discoloration in the form of strips, fins: congestion at the base of fins, or even hemorrhages, abdomen: slightly distended in some cases, anal opening: congested protruded anal opening) were collected from different locations in Egypt (River Nile in Giza, Kafr Elzayat, Bani-sweif and a breeding farm at El-Tal El-Kebir) during the period 2013–2014. Selection of locations and farms from which the diseased fish were collected depended on their availability at time sampling, accessibility and proximity. After collection, the sampled fish were placed in expanded polystyrene boxes, covered with a plastic film, transported refrigerated to the laboratory and processed for microbiological analysis within 3 h in order to detect *Streptococcus* spp.. Septicaemic fish samples were submitted to the Fish Disease Department, Animal Health Research Institute, Dokki, Egypt.

### Isolation and phenotypic characterization of bacteria

The swabs from the fish matrix that were collected (liver, kidney, brain, spleen and ascitic fluid) were immediately streaked onto Columbia agar supplemented with 5% defibrinated sheep blood (CBA) (Oxoid Ltd., Basingstone, England) plates and incubated at 30 °C for 24 h in 5% CO2 atmosphere. Suspected colonies were picked, purified and subjected to phenotypic differential analysis by conventional procedures by their morphological, physiological and biochemical plate and tube tests and in addition to the Gram-positive identification (GPI) cards of the Vitek 2 system (bioMérieux, France) which was used following the manufacturer’s instructions. The ability of the strains to grow at 10 and 45 °C was tested in Brain-heart infusion medium over a period of 10 days.

### Preparation of genomic DNA and genetic characterization

The suspected *Streptococcus* isolates were cultured in Todd Hewitt Broth at 25 C for 24 h, and genomic DNA was extracted using the Qiagen Genomic-tip 500/G kit (Qiagen, Hilden, Germany) according to the manufacturer’s instructions. DNA concentration was estimated spectrophotometrically before use. The extracted DNA was used in the process of amplification of the 16S rRNA gene of the *Streptococcus* species -specific isolates with a pair of specific primers for each species using polymerase chain reaction (PCR) as outlined in Table 1. Seven μl of the amplified products was electrophoresed using 1.0% (*w*/*v*) agarose gel in 1× TBE electrophoresis buffer (0.1 mM Tris/HCI, 0.1 mM boric acid, 0.002 mM EDTA, pH 8.3). The gel was stained with GelRed Nucleic Acid Gel Stain. A negative control (no template DNA) and positive controls consisting of *E. gallinarum* ATCC 49673, *E. faecalis* ATCC 19433, *L. garvieae* ATCC 43921, *S. pluranimalium* ATCC 700864^T^, *A. viridans* ATCC 11536 T (accession no. M58797), *S. anginosus* ATCC33397 were included in each run.

**Table 1:** Oligonucleotide primers sequences and size of the PCR-targeted products

Microorganism	Target Gene	Bp fragment	Primer sequence (5′ - 3′)	Annealing Temp (°C)	References
*E. faecalis / E. gallinarum*	16S rRNA (Genus -specific primers)	112 bp	F- TAC TGA CAA ACC ATT CAT GAT G	59 (*E. faecalis*) 50 (*E. gallinarum*)	[23]
			R- AAC TTC GTC ACC AAC GCG AAC		
*E. faecalis*	*ddl*_*E.fecalis*_ (Species-specific primers)	941 bp	F- ATC AAG TAC AGT TAG TCT	55	[24]
			R- ACG ATT CAA AGC TAA CTG		
*L. garvieae*	16S rRNA (species-specific primers)	1100 bp	F- CAT AAC AAT GAG AAT CGC	58	[25]
			R- GCA CCC TCG CGG GTTG		
*S. pluranimalium*	16S rRNA (species-specific primers)	1500 bp	F- AGA GTT TGA TCC TGG CTC AG	52	[26]
			R- ACG GCT ACC TTG TTA CGA CTT		
*A. viridans*	16S rRNA gene (species-specific primers)	540 bp	F- GTG CTT GCA CTT CTG ACG TTA GC	55	[27]
			R- TGA GCC GTG GGC TTT CAC AT		
*S. anginosus*	MIL (*pbp2b*) (Anginosus Group specific)	275 bp	F- TGC TGC AAC GGT AGC TAA TGG	58	[28]
			R- GAA AGG TTT CTG CTG TCC CTG		
	16srRNA (Species-specific primers)	105 bp	F- GCG TAG GTA ACC TGC CTA TTA GA	58	
			R- CGC AGG TCC ATC TAC TAGC		

### Serological assays

The Lancefield streptococcal group of the isolated strains was identified using the Oxoid Streptococcal Grouping Kit DR0585 (Oxoid Ltd., Basingstone, England) following the manufacturer’s instructions.

### Antimicrobial susceptibility testing

The resistance of the isolated strains to antibiotics was assayed by the disc diffusion method [18, 29–31]. *E. faecalis* ATCC 29212, *Escherichia coli* ATCC 25922 and *Aeromonas salmonicida* subsp. *salmonicida* ATCC 33658, *Pseudomonas aeruginosa* ATCC 27853 were used as Quality control strains for disk diffusion susceptibility testing of aquatic bacterial isolates [32]. The upcoming antibiotic discs (Oxoid, Ltd., Basingstoke, England) selected for testing are the most frequently used and prescribed in Egypt which are inhibitors of protein synthesis: phenicols (chloramphenicol), tetracyclines (tetracycline) and macrolides (erythromycin) for both *Enterococcus* and *Streptococcus* species, while aminoglycosides (gentamicin, streptomycin and amikacin) only for *Enterococcus* species; cell wall synthesis: penicillins (penicillin, ampicillin, amoxicillin-clavulonic acid and pipercillin-tazobactum) and glycopeptides (vancomycin) for both *Enterococcus* and *Streptococcus* species and polymyxins (colistin) for *Enterococcus* species; nucleic acid synthesis: ansamycins (rifampicin) and nitrofurantoins (nitrofurantoin) were used for both *Enterococcus* and *Streptococcus* species, fluoroquinolones (ciprofloxacin for *Enterococcus* species and ofloxacin for *Streptococcus* species), quinolones (nalidixic acid for *Enterococcus* species) and lincosamides (clindamycin) for *Streptococcus* species.

### Statistical analysis

The experimental data obtained was subjected to multiple linear regressions using Microsoft Excel 2007 to evaluate the statistical analysis between the percentage of antimicrobial resistance (y- range) and actual concentration (x- axis) for all antibiotics to enable us to plot percent inhibition versus drug concentration for each strain*.*

## Results

### Biochemical and molecular characterization

The microbiological cultures on CBA from the swabs taken from the internal organs (kidney, spleen and brain) and ascitic fluid revealed the presence of pinpoint white colonies (0.5 mm Ø) in pure culture or as major colony type. The Gram-stained smears that were taken from the presumptive *Streptococcus* colonies revealed Gram-positive chain forming cocci (0.6–0.9 μm Ø) small or medium sized in diploids, short chain, chain in the form of Y / V-shape, or in long chain, β-haemolytic, catalase and aesculin hydrolysis test negative, ability to grow at 25 and 37 °C and at pH 9.6 in the absence of 6.5% NaCl or 40% bile salts. Table 2 illustrates the effect of incubation temperature and NaCl 6.5% and bile salts on the growth of different isolates their role in identification of different types of isolates. The seventeen isolates were identified using Vitek 2 compact through the biochemical characterization of different isolates.

**Table 2:** Differential diagnosis between different bacterial species using growth conditions

Isolate	Growth at		Growth on 6.5% NaCl BHI agar			Growth at 40% Bile Salt BHI agar
	10^ͦ^ C	45^ͦ^ C	After 24 h	After 48 h	After 72 h	After 7 days
*Aerococcus viridans*	After 48 h (very faint) but good after 72 h	+	No growth	Very weak growth	Weak growth	No growth
*E. faecalis*	after 48 h	+	Very weak growth	Good growth	Very good growth	No growth
*Lactococcus garvieae*	after 48 h (very faint) but good after 72 h	+	No growth	Very weak growth	Very good growth	No growth
*S. pluranimalium*	After 48 h	+	very weak growth	Very weak growth	Very good growth	No growth
*E. gallinarum*	After 48 h (very faint) but good after 72 h	_	No growth	No growth	No growth	No growth
*S. dysgalactiae* subspecies *equisimilis*	No growth	+	No growth	No growth	No growth	No growth
*S. anginosus*	No growth	No growth	No growth	No growth	No growth	No growth

None of our 16 isolates could be serologically typed to any Lancefield group and in the Vitek 2 inconclusive results were obtained. The only result obtained was with the *S. dysgalactiae* subspecies *equisimilis* (one isolate) which was grouped in the C group.

Analysis of the 16S rRNA gene confirmed that the 17 tilapia isolates studied were 6/17 *E. faecalis,* 2/17 *E. gallinarum,* 3/17 *S. pluranimalium,* 2/17 *A. viridans,* 1/17 isolate of each *S. dysgalactiae, S. anginosus, L. garvieae* and *Granulicatella elegans.*

### Prevalence and identification of *Enterococcus*, *Lactococcus* and *Streptococcus* species isolated from diseased fish

Overall, 17 *Streptococcus* species were collected and the *Streptococcus* spp*.* occurred in 8/80 (10%) liver samples, 4/80 (5%) spleen samples, in 3/80 (3.8%) kidney samples and 1/80 (1.3%) brain and ascitic fluid samples each. The logistic regression showed that the recovery of *Streptococcus* spp. was significantly affected by fish matrix (*P* < 0.001). The test of independence showed a significant association between the recovery of *Streptococcus* spp. from a given matrix and the location (Table 3).

**Table 3:** Prevalence and Types of *Enterococcus, Lactococcus* and *Streptococcus* species isolated from diseased fish organs using 16 s rRNA

Matrix	Total No.	Species																	
		*E. faecalis*		*S. pluranimalium*		*A. viridans*		*E. gallinarum*		*S. anginosus*		*L. garvieae*		*S. dysgalactiae equisimilis*		*Granulicetella elegans* / *Leuconostoc mesenteroides cremoris*		Total	
		n	%	n	%	n	%	n	%	n	%	n	%	n	%	n	%	n	%
Liver	80	3	3.8	2	2.5	1	1.3	1	1.3	1	1.3	1	1.3	0	0	0	0	8	10.0
Kidney	80	1	1.3	0	0	1	1.3	1	1.3	0	0	0	0	0	0	0	0	3	3.8
Brain	80	0	0	0	0	0	0	0	0	0	0	0	0	1	1.3	0	0	1	1.3
Spleen	80	1	1.3	1	1.3	0	0	1	1.3	0	0	0	0	0	0	1	1.3	4	5
Ascitic fluid	3	1	33.3	0	0	0	0	0	0	0	0	0	0	0	0	0	0	1	33.3
Total	323	6	1.9	3	0.9	2	0.6	2	0.9	1	0.3	1	0.3	1	0.3	1	0.3	17	5.3

n, number of isolates

The rate of bacteria isolated from the diseased tilapia fish, were isolated in a rate of 21.25%. *E. faecalis* showed the highest number of isolates (6 isolates) in a rate of 7.5%, followed by *S. pluranimalium* (3.75) (Table 3). *E. faecalis* was the most predominant isolate (6/80; 1.9%) and the liver was the most prominent site for the infection with *E. faecalis* (3.75%). Also, *E. faecalis* was the sole isolate from the ascitic fluid (1.25%). *S. pluranimalium* was the second predominant isolate (0.93%) being also predominantly localized in the liver (2.5%) and spleen (1.3%). Similarly, *E. gallinarum* was isolated at a rate of 1.3% from the liver and kidney. *S. dysgalactiae* subspecies *equisimilis* was only isolated from the brain (1.25%).

### Antimicrobial susceptibility

**Fig. 2:**
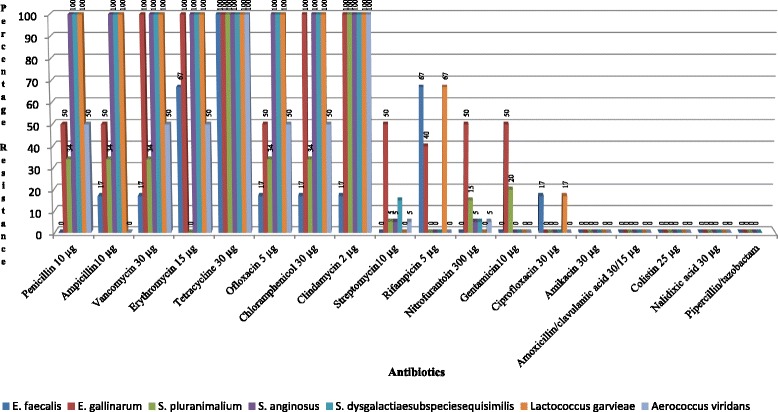
Percentage resistance of the seven species of streptococci isolated from Nile tilapia (*Oreochromis niloticus*) to the 18 antibiotics tested

The susceptibility of 17 *Streptococcus* strains was assessed against 18 different antibiotics (Fig. 2). Highest resistant to Tetracycline 94.1% (16/17) the lowest resistance was observed for Nitrofurantoin, Streptomycin, Gentamicin and Trimethoprim/Sulphamethaxozol 5.9% (1/17 each). The 17 isolates showed total susceptibility to Amoxicillin/Clavulanic acid, Piperacillin/Tazobactam, Nalidixic acid, Colistin and Amikacin. There was no significant relationship between the percentage of antimicrobial resistance (y- range) and actual concentration (x- axis) for all combined antibiotics and consequently no regression line could be drawn (Table 4).

**Table 4:** Statistical analysis between the percentage of antimicrobial resistance (y- range) and actiual concentration (x- axis) of antibiotics for the isolated seven strains

	*E. faecalis*	*E. gallinarum*	*S. pluranimalium*	*S. anginosus*	*S. dysgalactiae subspecies equisimilis*	*L. garvieae*	*A. viridans*	
*E. faecalis*	1							
*E. gallinarum*	0.534252	1						
*S. pluranimalium*	0.434469	0.696294	1					
*S. anginosus*	0.458603	0.7774	0.693549	1				
*S. dysgalactiae subspecies equisimilis*	0.455604	0.783461	0.692309	0.999059	1			
*L. garvieae*	0.593849	0.767848	0.650752	0.953986	0.950886	1		
*A. viridans*	0.555896	0.785501	0.873946	0.824254	0.823841	0.784558	1	

Multiple resistance was observed in 16/17 strains (94.1%). In strains with multiple resistance, the most frequent antibiotic combination was penicillin, ampicillin, vancomycin, chloramphenicol, rifampicin, ofloxacin, clindamycin, erythromycin and tetracycline (7/17, 41.2%) representing eight classes (Table 5).

**Table 5:** Antibiotic resistance combinations profile of the 17 *Streptococcus* species isolated from Nile tilapia

Antibiotic resistance combination profile	number of isolates/17	% of isolates	number of antibiotics	Number of classes
P, Am	1	5.9	2	1
P, Am,VA	1	5.9	3	2
P, Am,VA, C	2	43	4	3
P, Am,VA, C, RD	1	5.9	5	4
P, Am,VA, C, RD, OFX	1	1.9	6	5
P, Am,VA, C, RD, OFX, DA	1	5.9	7	6
P, Am,VA, C, RD, OFX, DA, E	3	17.6	8	7
P, Am,VA, C, RD, OFX, DA, E, TE	7	41.2	9	8

*P* penicillin, *Am* ampicillin, *Va* vancomycin, *C* chloramphenicol, *RD* rifampicin, *OFX* ofloxacin, *DA* clindamycin, *E* erythromycin, *TE* tetracycline

## Discussion

In this work, a collection of 17 strains of *Streptococcus* species isolated from diseased tilapia were biochemically, serologically and genetically characterized and data on their prevalence of the disease in Egypt. In contrast to other results [18], 16 of our isolates could not be serologically typed to any Lancefield group and in the Vitek 2 inconclusive results were obtained, a phenomenon which was also previously observed by Figueiredo et al. [33]. On the other hand, our sole isolate of *S. dysgalactiae* subspecies *equisimilis* which was grouped in the C group was also identified in Japan by Nomoto et al. [34, 35] and Abdelsalam et al. [36], in the amberjack (*Seriola dumerili*) and yellowtail (*Seriola quinqueradiata*) and in Amur sturgeon (*Acipenser schrenckii*) in China [37] and in Nile tilapia (*Oreochromis niloticus* (L.) in Brazil [20].

Our detection of *Streptococcus* species in the three organ tissues (liver, spleen and kidney) implicates them as the target organs of the organism [38–40]. Fish pathogenic strains have been described, as either an α- or ß-haemolytic or as non-haemolytic [41]. Superficially, this data could surmise heterogeneity amid the pathogens, inspite of the fact that some confirmed ranks such as *S. agalactiae*, incorporates both α– and ß-haemolytic strains. It should be noted that, many features are common within the predominance of fish pathogens [9]. Yet, this does not exclude some discrepancies reported in isolates recovered from the Transvaal in South Africa [42] and other isolates from Japan [43–45]. Such discrepancies could be due to the dissimilarity and absence of a standardized test protocols and/or point to heterogeneity in the species composition of the organisms.

The development and application of molecular methodology has been of great benefit and advantage through facilitating identification precision, classification of streptococci [17] and time-saving. Thus, our use of the PCR in the species -specific analysis of the 16S rRNA gene was of great assistance in the present investigation and unequivocally demonstrated that all of them belonged to the *Streptococcus* species. To a certain degree, geographical discrepancies have been reported from South Africa [42], Japan [46] and Italy [47]. It is of interest to reveal that according to the available literature, *L. garvieae* although being rarely isolated from tilapia with streptococcosis, it was identified in two countries only, Brazil [19] and Indonesia [10]. This makes our detection of *L. garvieae* in our isolates, the third report. Interestingly, the isolation of *Leuconostoc mesenteroides cremoris* in our investigation was not reported before from tilapia.

Aquaculture is a developing field where antibiotics are intensely used, either specifically to the water or in fish sustenance, as prophylactics to control infections, which has brought about the blooming of AR genes in the aquatic environment [48]. The measure of AMR in aquaculture is under dispute as there is an absence of consent on a standard indicator organism. Ratably, the utilization of antimicrobials in aquaculture seems, by all accounts, to be of substantially more prominent greatness when compared with terrestrial animals. A shortage of solid ratable and subjective information on antimicrobial use (AMU) in different animal production systems over the area is a noteworthy hole in the exploration. Subjective and ratable accessible information (for the most part AMU recurrence of particular antimicrobials) from particular examinations recommends a high assorted variety of antimicrobials utilized both as development promoters (AGPs), and in addition for prophylactic and treatment purposes, despite the fact that outcomes are hard to look at crosswise over investigations. As a result of the extensive contrasts in underway frameworks, sampling and assay techniques, phenotypic evaluations of AMR pervasiveness in this audit should be deciphered with extraordinary care. Generally, the collection of samples are liable to have an expansive testing mistake. The antibiotics used in aquaculture represent different classes of antibiotics, some of which are classified as “Highest Priority Critically Important Antimicrobials” in medical treatment in the WHO list of Critically important antimicrobials for human medicine (CIA list) such as fluoroquinolones, 3rd and 4th Generation cephalosporins, Macrolides and ketolides and Glycopeptides [49]. Fortunately, the isolated strains in the present investigation were not resistant to any of the CIA list with the exception of erythromycin. Globally, the use of antimicrobial agents is regulated differently from country to country, being either very strict or under-regulated. Possible hazards associated with drug abuse in fish farming are the presence of residues in food and the development of antibiotic resistance in the bacterial population. In agreement, to our surprise numerous multi-ABR- bacteria (49%) were observed among our isolates, irrespective of their traditional resistance to beta lactam antibiotics [50, 51]. It was theorized that, resistance to these antibiotics could be a result of their overuse or/and misuse in the aquaculture industry, could arise from gene mutations or by acquisition of transferable genetic elements such as integrons [50, 52]. The property of horizontal spread of resistance genes was incriminated to be responsible for the recorded levels of multi-resistance. Notwithstanding AMU in aquaculture, the basic routine with regards to releasing fertilizer from earthbound creatures into water frameworks leaves the aquatic environments especially powerless against the improvement of AMR.

## Conclusion

Worldwide death of fish caused by streptococcal infection remains an extortionate issue for growers of Nile tilapia and other warmwater fish, yet can be handled effectively with a cohesive, supportable scheme incriminating vaccines and new-generation antibiotics developed specifically for aquaculture. Strains of *Streptococcus* associated with infection in humans can cause people handling affected whole raw fish, primarily tilapia, to become at risk of developing neonatal meningitis and sepsis (early-onset and late-onset disease), cellulitis, subdural empyema, endocarditis or arthritis following a puncture wound [53–55]. Substantially, our results highlight the necessity to encourage conscientious fish producers, efficient husbandry performances and judicious practise of antibiotics in aquaculture. It should be emphasized that, *S. dysgalactiae* has emerged as a causative agent in fish disease, with a highly medical significance in mammalian and human health as alpha-hemolytic Lancefield group C *S. dysgalactiae* of fish origin, was implicated in ascending upper limb cellulitis in humans [56].

## Abbreviations

AGPs: Antimicrobial growth promoters
AMU: Antimicrobial usage
CBA: Columbia blood agar
CIA: Central Intelligence Agency
FAO: Food and Agriculture Organization
GPI: Gram-positive identification
MT: Metric tons
USD: United States Dollar
WHO: World Health Organization

## References

[1] Jamie Allen's. Family tree & ancient genealogical allegations version 77, 2016. The PEDIGREE of Nile tilapia.

[2] Trewaves E (1983). Tilapia fishes of the genera Sarotherodon.

[3] Balarin JD, Hatton JP (1979). Tilapia: A guide to their biology and culture in Africa.

[4] Pillay TVR (1990). Aquaculture principles and practices. Fishing news books.

[5] FAO. Food and Agriculture Organization of the United Nations. Fishstat plus. FAO. Rome 2004.

[6] Babylon Software Ltd. Babylon’s 10 online Dictionary & Translation, Copyright © 2014–2016.

[7] Food FAO. Agriculture Organization of the United Nations. Yearbook 2010, fishery and aquaculture statistics. Rome. 2012;

[8] FAO. Fisheries and Aquaculture Department. About us - Fisheries and Aquaculture Department. In: FAO Fisheries and Aquaculture Department [online]. Rome. Updated. [Cited 15 September 2016]. http://www.fao.org/fishery/about/en

[9] Austin B, Austin DA. Bacterial fish pathogens: disease of farmed and wild fish, fifth edition ISBN 978–94–007-4883-5 ISBN 978–94–007-4884-2 (eBook) springer Dordrecht Heidelberg new York London Library of Congress control number: 2012946925 © springer science+business media Dordrecht 2012.

[10] Anshary H, Kurniawan RA, Sriwulan S, Ramli R, Baxa DV (2014). Isolation and molecular identification of the etiological agents of streptococcosis in Nile tilapia (*Oreochromis niloticus*) cultured in net cages in Lake Sentani, Papua, Indonesia. Springer Plus.

[11] Klesius PH, Shoemaker CA, Evans JJ. *Streptococcus*: a worldwide fish health problem. 8th international symposium on tilapia in aquaculture. Cairo. 2008:83–107.

[12] Yanong RPE, Francis-Floyd R. Streptococcal Infections of Fish. This document is Circular 57, one of a series of the Fisheries and Aquatic Sciences Department, Florida Cooperative Extension Service, Institute of Food and Agricultural Sciences, University of Florida. Original publication date April 2002. Revised August 2006. Reviewed June 2013. Visit the EDIS website at http://edis.ifas.ufl.edu 2013.

[13] Siti-Zahrah A, Padilah B, Azila A, Rimatulhana R, Shahidan R, Bondad-Reantaso MG, Mohan CV, Crumlish M, Subasinghe RP (2008). Multiple streptococcal species infection in cage-cultured red tilapia but showing similar clinical signs, pp. 313–320. Diseases in Asian Aquaculture VI.

[14] Amal MNA, Zamri-Saad M (2011). Streptococcosis in tilapia (*Oreochromis niloticus*): a review. Pertanika J Trop Agric Sci.

[15] Li YW, Liu L, Huang PR, Fang W, Luo ZP, Peng HL (2014). X chronic streptococcosis in Nile tilapia, *Oreochromis niloticus* (L.), caused by *Streptococcus agalactiae*. J Fish Dis.

[16] Sudheesh, P. S., Al-Ghabshi, A., Al-Mazrooei, N., and Al-Habsi, S. (2012). Comparative Pathogenomics of bacteria causing infectious diseases in fish. Inter J Evol biol. 2012;2012:457264. doi:10.1155/2012/457264. 10.1155/2012/457264 PMC3364575 22675651

[17] Toranzo AE, Magarin B, Romalde JL. A review of the main bacterial fish diseases in mariculture systems. Aquaculture. 2005;246:37–61. doi:10.1016/j.aquaculture.2005.01.002

[18] Romalde JL, Ravelo C, Vald́es I, Magariños B, de la Fuente E, Martín CS (2008). *Streptococcus phocae*, an emerging pathogen for salmonid culture. Vet Microbiol.

[19] Evans JJ, Klesius PH, Shoemaker CA (2009). First isolation and characterization of *Lactococcus garvieae* from Brazilian Nile tilapia, *Oreochromis niloticus* (L.) and pintado, *Pseudoplathystoma corruscans* (Spix & Agassiz). J Fish Dis.

[20] Netto LN, Leal CA, Figueiredo HC (2011). *Streptococcus dysgalactiae* as an agent of septicaemia in Nile tilapia, *Oreochromis niloticus* (L.). J Fish Dis.

[21] Hoshina T, Sano T, Morimoto Y (1958). A *Streptococcus* pathogenic for fish. J Tokyo Univ Fisher.

[22] Ghittino C. La estreptococosis en los peces. Aquatic 2, art. 1999;605. Available at URL http://aquatic.unizar.es/n2/art605/lact_rev.htm.

[23] Karsidani SH, Soltani M, Nikbakhat-Brojeni G, Ghasemi M, Skall H (2010). Molecular epidemiology of zoonotic streptococcosis/lactococcosis in rainbow trout (*Oncorhynchus mykiss*) aquaculture in Iran. Iran J Microbiol.

[24] Biavasco F, Foglia G, Paoletti C, Zandri G, Magi G, Guaglianone E (2007). VanA-type enterococci from humans, animals, and food: species distribution, population structure, Tn*1546* typing and location, and virulence determinants. Appl Environ Microbiol.

[25] Fadaeifard F, Momtaz H, Rahimi E, Mirzakhani A (2012). Detection of *Streptococcus iniae* and *Lactococcus garvieae* by multiplex polymerase chain reaction (PCR) in some rainbow trout farms of Iran. Afr J Biotechnol.

[26] Wan L, Chen SJ, Wang CD, Li DS, Zheng Z, Wang CD (2012). Identification and genotypic analysis of *Streptococcus* spp. isolated from Giant pandas in China by PCR-based methods. Afr J Microbiol Res.

[27] Martin V, Vela AI, Gilbert M, Cebolla J, Goyache J, Domínguez L (2007). Characterization of *Aerococcus viridans* isolates from swine clinical specimens. J Clin Microbiol.

[28] Takao A, Nagamune H, Maeda N. Identification of the anginosus group within the genus *Streptococcus* using polymerase chain reaction*.* FEMS Microbiol Lett 2004;233:83–89. doi:10.1016/j.femsle.2004.01.042. 10.1016/j.femsle.2004.01.042 15043873

[29] Channaiah LH, Subramanyam B, McKinney LJ, Zurek L (2010). Stored-product insects carryantibiotic-resistant and potentially virulent enterococci. FEMS Microbiol Ecol.

[30] CLSI. The Clinical and Laboratory Standards Institute (2014). Methods for Antimicrobial Disk Susceptibility Testing of Bacteria Isolated From Aquatic Animals. Approved guideline (VET03-a).

[31] Iweriebor BC, Gaqavu S, Obi LC, Nwodo UU, Okoh AI (2015). Antibiotic susceptibilities of *Enterococcus* species isolated from hospital and domestic wastewater effluents in Alice, eastern Cape Province of South Africa. Int J Environ Res Public Health.

[32] Miller RA, Walker RD, Baya A, Clemens K, Coles M, Hawke JP (2003). Antimicrobial susceptibility testing of aquatic bacteria: quality control disk diffusion ranges for *Escherichia coli* ATCC 25922 and *Aeromonas salmonicida* subsp. *salmonicida* ATCC 33658 at 22 and 28°C. J Clin Microbiol.

[33] Figueiredo HCP, Netto LN, Leal CAG, Pereira Ulisses P, Mian Glaúcia F (2012). *Streptococcus iniae* outbreaks in Brazilian Nile tilapia (*Oreochromis niloticus* L.) farms. Braz J Microbiol.

[34] Nomoto R, Munasinghe LI, Jin DH, Shimahara Y, Yasuda H, Nakamura A (2004). Lancefield group C *Streptococcus dysgalactiae* infection responsible for fish mortalities in Japan. J Fish Dis.

[35] Nomoto R, Unose N, Shimahara Y, Nakamura A, Hirae T, Maebuchi K (2006). Characterization of Lancefield group C *Streptococcus dysgalactiae* isolated from farmed fish. J Fish Dis.

[36] Abdelsalam M, Chen SC, Yoshida T (2010). Phenotypic and genetic characterizations of *Streptococcus dysgalactiae* strains isolated from fish collected in Japan and other Asian countries. FEMS Microbiol Lett.

[37] Yang W, Li A (2009). Isolation and characterization of *Streptococcus dysgalactiae* from diseased *Acipenser schrenckii*. Aquaculture.

[38] Eldar A, Bejerano Y, Livoff A, Horovitcz A, Bercovier H (1995). Experimental streptococcal meningo-encephalitis in cultured fish. Vet Microbiol.

[39] Lahav D, Eyngor M, Hurvitz A, Ghittino C, Lublin A, Eldar A (2004). *Streptococcus iniae* type 11 infections in rainbow trout *Oncorhynchus mykiss*. Dis Aquatic Org.

[40] Abuseliana AF, Daud HHM, Abdul Aziz S, Bejo SK, Alsaid M (2011). Pathogenicity of *Streptococcus agalactiae* isolated from a fish in Selangor to juvenile red tilapia (*Oreochromis* sp.). J Anim Vet Adv.

[41] Murray PR, Baron EJ, Jorgensen JJ (2003). *Streptococcous* general methods in: manual of ClinicalMicrobiology.

[42] Boomker J, Imes GD, Cameron CM, Naude TW, Schoonbee HJ (1979). Trout mortalities as a result of a Streptococii infection. Onderstepoort J Vet Res.

[43] Minami T, Nakamura M, Ikeda Y, Ozak HA. Beta-hemolitic *Streptococcus* isolated from cultured yellowtail. Fish Pathol*.* 1979;14:15–9. doi:10.3147/jsfp.14.33

[44] Kitao T, Aoki T, Sakoh R (1981). Epizootic caused by b-haemolytic *Streptococcus* species in cultured freshwater fish. Fish Pathol..

[45] Ugajin M. Studies on *Streptococcus* sp as a causative agent of an epizootic among the cultured ayu (*Plecoglossus altivelis*) in Tochigi prefecture, Japan, 1980. Fish Pathol. 1981;16:119–27. doi:10.3147/jsfp.16.119

[46] Kusuda R, Komatsu I, Kawai K (1978). *Streptococcus* sp. isolated from an epizootic of cultured eels. Nippon Suisan Gakkaishi.

[47] Ghittino C, Prearo M (1992). Report of streptococcosis in rainbow trout (*Oncorhynchus mykiss*) in Italy: preliminary note. Boll Soc Ital Patalog Ittica.

[48] Heuer OE, Kruse H, Grave K, Collignon P, Karunasagar I, Angulo FJ (2009). Human health consequences of use of antimicrobial agents in aquaculture. Clin Infect Dis.

[49] WHO. The World Health Organization. WHO fact sheets on food safety: Highest Priority Critically Important Antimicrobials, 2016.

[50] Defoirdt T, Sorgeloos P, Bossier P (2011). Alternatives to antibiotics for the control of bacterial disease in aquaculture. Curr Opin Microbiol.

[51] Di Cesare A, Luna GM, Vignaroli C, Pasquaroli S, Tota S, Paroncini P (2013). Aquaculture can promote the presence and spread of antibiotic-resistant enterococci in marine sediments. PLoS One.

[52] Huddleston JR (2014). Horizontal gene transfer in the human gastrointestinal tract: potential spread of antibiotic resistance genes. Infect Drug Resis.

[53] MMWR (1996). Invasive infection with *Streptococcus iniae*, Ontario, 1995–1996. Morb Mort Weekly Rep.

[54] Weinstein MR, Litt M, Kertesz DA, Wyper P, Rose D, Coulter M (1997). Invasive infections due to a fish pathogen, *Streptococcus iniae* study group. New Eng J Med.

[55] Aryasinghe L, Sabbar S, Kazim Y, Awan LM, Khan HK (2014). Streptococcus *pluranimalium*: a novel human pathogen?. Inter J Surg Case Rep.

[56] Koh TH, Sng LH, Yuen SM, Thomas CK, Tan PL, Tan SH (2009). Streptococcal cellulitis following preparation of fresh raw seafood. Zoonoses Public Health.

